# Genome-Wide Analysis of Circular RNAs Reveals circCHRNG Regulates Sheep Myoblast Proliferation via miR-133/SRF and MEF2A Axis

**DOI:** 10.3390/ijms232416065

**Published:** 2022-12-16

**Authors:** Yue Liu, Qian Chen, Jingjing Bao, Yabin Pu, Jianlin Han, Huijing Zhao, Yuehui Ma, Qianjun Zhao

**Affiliations:** 1 Institute of Animal Science, Chinese Academy of Agricultural Sciences, Beijing 100193, China; 2 CAAS-ILRI Joint Laboratory on Livestock and Forage Genetic Resources, Institute of Animal Science, Chinese Academy of Agricultural Sciences (CAAS), Beijing 100193, China; 3 International Livestock Research Institute (ILRI), Nairobi 00100, Kenya

**Keywords:** skeletal muscle, circRNA, RNA-seq, circCHRNG, ceRNA

## Abstract

As relatively new members of the non-coding RNA family, circRNAs play important roles in a variety of biological processes. However, the temporal expression pattern and the function of circRNAs during sheep skeletal muscle development remains unclear. This study aimed to identify circRNAs related to sheep skeletal muscle development and explore their roles in myoblast proliferation. The circRNA expression profiles of longissimus dorsi of sheep from F90, L30, and A3Y were obtained by the RNA-seq method. The function and mechanisms of the novel circCHRNG in muscle satellite cell proliferation were explored using CCK-8 assay, Western blot, qPCR, and dual-luciferase reporter assay. We identified 12,375 circRNAs, including 476, 133, and 233 DEcircRNAs found among three comparative groups. KEGG results showed that DEcircRNAs were enriched in muscle contraction, the regulation of cell proliferation, and the AMPK, insulin, and PI3K-Akt signaling pathways. Notably, a novel circRNA, termed circRNA CHRNG, acts as a miR-133 sponge to promote skeletal muscle satellite cell proliferation. Our study provides a systematic description of circRNAs of ovine skeletal muscle across fetal, lamb, and adult stages. GO and KEGG analyses showed that DEcircRNAs were enriched in multiple pathways associated with muscle development, such as the PI3K-Akt and AMPK signaling pathways. In addition, we propose that circCHRNG acts as a miR-133 sponge to upregulate the expression levels of SRF and MEF2A, thereby promoting myoblast proliferation.

## 1. Introduction

Circular RNA (circRNA) is a relatively new member of the non-coding RNA family [1,2] and plays important roles in a variety of biological processes, such as growth, development, and disease [3,4]. Studies have shown that circRNAs are specially spliced circle RNAs, which are resistant to exonuclease RNase R [5]. CircRNAs are predominantly generated by back-splicing reactions, in which the 5′ and 3′ ends of linear RNAs are directly ligated, generally consisting of exons and introns [6]. According to the structure formed, circRNAs are divided into four types: exon circular RNAs (ecircRNAs), circular intronic RNAs (ciRNAs), exon–intron circular RNAs (EICiRNAs), and tRNA intronic circular RNAs (tricRNAs) [7]. Recent studies have found that circRNAs exert biological functions in multiple ways. As non-coding RNAs, circRNAs not only serve as microRNA and RNA-binding protein sponges [8] but directly act as translation templates [9,10]. In addition, circRNAs can modulate the transcription of the parent genes [11]. Currently, most research has demonstrated that circRNAs act as miRNA sponges competing with targeted mRNAs and thus regulate the expression of the miRNA target gene [12].

Skeletal muscle is the most important component of animal body mass and directly affects meat quantity and quality. Skeletal muscle growth and development is a complex and orchestrated biological process, which is regulated by a variety of genes, transcription factors, and ncRNAs [13]. Accumulating evidence indicates that circRNAs are involved in skeletal muscle growth and development in many species, such as pigs and chickens [14,15,16]. For example, circRBFOX2 was identified to promote chicken myoblast proliferation as a sponge of miR-206 [16]. It has been reported that circRNAs are involved in skeletal muscle growth and development [17], in which circRNAs are abundant and dynamically expressed [17,18]. The expression pattern and potential function of circRNAs of sheep skeletal muscle are still largely unknown. Therefore, it is necessary to investigate the expression dynamics of the circRNAs and the corresponding mechanism underlying the regulation of the sheep muscle development.

In this study, to systematically investigate the expression profile and function of circRNAs of sheep skeletal muscle, we performed high-throughput RNA sequencing for longissimus dorsi muscle of a 90-day-old fetus (F90), 30-day-old lamb (L30), and three-year-old adult sheep (A3Y). We also predicted circRNAs’ potential function and constructed the comprehensive circRNA–miRNA interaction network. Notably, we focused on the functions of one of the most downregulated circRNAs, circ-023984, which was subsequently named circCHRNG according to its host gene CHRNG. However, the function and regulatory mechanism of circRNA CHRNG in muscle development remain unclear. Previous studies have reported that CHRNG (cholinergic receptor nicotinic gamma subunit) is the γ subunit and overexpression of CHRNG in bovine preadipocytes inhibits the proliferation and differentiation of bovine preadipocytes [19]. In addition, the gamma subunits promote neuromuscular signal transduction and are also important for neuromuscular histogenesis [20]. Mechanistically, circCHRNG acts as a miR-133 sponge indirectly to upregulate the expression of SRF and MEF2A, thereby inducing skeletal muscle proliferation. Our study not only provides a valuable transcriptional regulatory resource for understanding the mechanisms of sheep muscle development but also reveals new clues for identifying the role of circRNAs in myogenic differentiation.

## 2. Results

### 2.1. Identification of circRNAs during Sheep Skeletal Muscle Development

**Figure 1:**
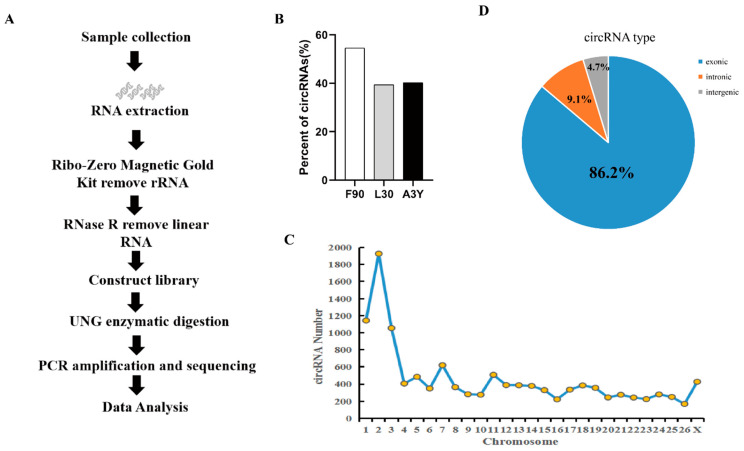
Identification of circRNAs of sheep longissimus dorsi muscle. (**A**) The workflow of circRNA analysis of skeletal muscle using RNA-seq method. (**B**) Proportion of circRNAs in each stage. (**C**) The distribution of circRNA on the chromosomes. (**D**) The type of the circRNA.

To investigate circRNA dynamic expression changes during sheep skeletal muscle growth, we performed RNA-seq analysis for longissimus dorsi in three periods using the strand-specific transcriptome sequencing method. The circRNA expression profiles of fetal sheep skeletal muscle (F90), lamb skeletal muscle (L30), and adult sheep skeletal muscle (A3Y) were analyzed (Figure 1A).

Each sample generated more than 10Gb of data. An average of 99.87% clean reads were mapped to the sheep reference genome (Oar_v4.0) (Table S3). A total of 12,357 circRNAs were identified and 5448 genes were annotated. We found that the number of circRNAs of F90 was obviously more than that of L30 and A3Y (Figure 1B). The mapping reads were uniformly distributed in each chromosome, and chromosome 2 had the largest number of circRNAs (Figure 1C). In our study, circRNAs were extensively transcribed from exon regions (86.2%), whereas a small fraction of circRNAs (<13%) originated from the exon–intron splicing junction and the intergenic and intron regions (Figure 1D), suggesting that circRNAs have the potential to regulate the expression of miRNA in a ceRNA manner. In summary, our result provided a comprehensive characterization of circRNAs during sheep muscle growth and development.

### 2.2. Identification of DEcircRNAs

**Figure 2:**
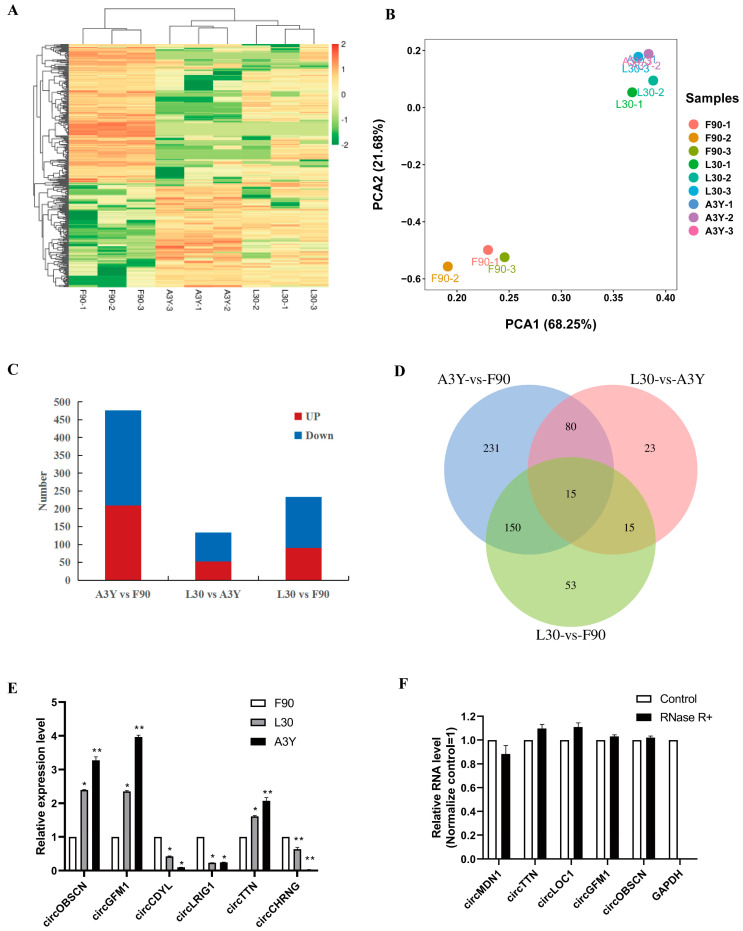
Analysis of differentially expressed circRNAs in sheep longissimus dorsi muscle. (**A**) Heatmap showing the expression dynamics of circRNAs in F90, L30, and A3Y. (**B**) PCA analysis. (**C**) DEcircRNA numbers. (**D**) DEcircRNA Venn diagram. (**E**) Validation of circRNA expression in sheep skeletal muscle by RT-qPCR. (**F**) Expression of circRNAs analyzed in RNA samples treated with both RNase R (–) and RNase R (+). We considered *p* < 0.05 to be statistically significant. * *p* < 0.05, ** *p* < 0.01.

To understand the expression changes of circRNAs in sheep muscle, we constructed circRNA expression profiles of the ovine longissimus dorsi muscle in the three developmental periods. Heatmap (Figure 2A) and PCA (Figure 2B) analysis showed that the circRNAs expression patterns of the L30 and A3Y groups were similar, but they were different from those of the F90 group. Furthermore, through differential expression analysis a total of 233 (L30 vs. F90), 133 (L30 vs. A3Y), and 476 (A3Y vs. F90) differentially expressed circRNAs (DEcircRNAs) were identified among the three groups (|log2FC| > 2, *p* < 0.05) (Figure 2C). Fifteen overlapped DEcircRNAs were observed in the three comparison groups (Figure 2D). Notably, L30 vs. A3Y had the fewest DEcircRNAs and F90 vs. A3Y contained the most DEcircRNAs, which indicated that the transcription differences between prenatal and postnatal muscle were dramatic.

To validate the sequencing data, we further experimentally detected the expression of circRNAs using the qPCR method. We chose the circRNAs mainly by considering the different expression patterns of the DEcircRNAs, including upregulated and downregulated circRNAs across three developmental stages. The qPCR result showed that the patterns of upregulation or downregulation of the six circRNAs were consistent with the sequencing results, suggesting that RNA-Seq data provided reliable information about the relative abundance of circRNAs (Figure 2E). We also tested thew resistance of circRNAs to RNase R digestion by real-time RT-PCR. All tested circRNAs were resistant to RNase R digestion, which confirmed their circular characteristics, whereas the linear mRNA of GAPDH was not detected (sensitive to RNase R) (Figure 2F).

### 2.3. Functional Enrichment Analysis of DEcircRNAs

**Figure 3:**
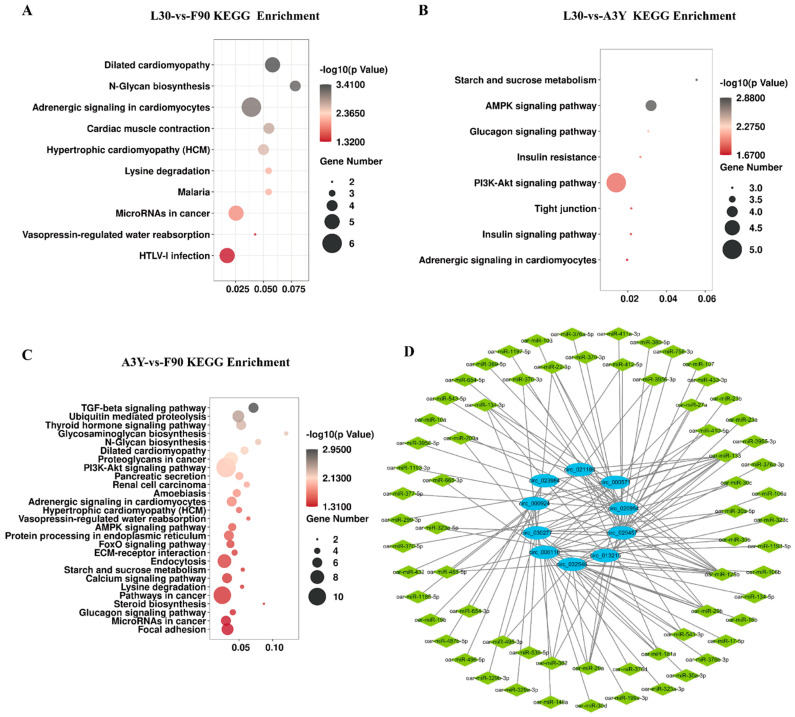
Kyoto Encyclopedia of Genes and Genomes pathways. (**A**) L30 vs. F90, (**B**) A3Y vs. F90, (**C**) L30 vs. A3Y. (**D**) Regulatory networks of circRNAs–miRNAs.

To explore the potential function of circRNAs, we performed GO enrichment analysis and KEGG pathway analysis of DEcircRNAs (|log2 FC| > 2, *p* < 0.01) in L30 vs. F90, L30 vs. A3Y, and A3Y vs. F90. The GO enrichment analysis suggested that circRNAs have different regulatory functions at different developmental stages (Figure S1). During muscle development, the upregulated DEcircRNAs were mainly enriched in pathways related to tissue development and energy metabolism. For example, for the L30-vs-F90 group, the upregulated DEcircRNAs were enriched in the pathway which were related to constituent of muscle and protein homodimerization activity. For the L30-vs-A3Y group, the upregulated DEcircRNAs were enriched in epithelial cell development and transcription coregulator activity, while the downregulated circRNAs were involved in pathways related to cell cycle (Figure S1). The enriched KEGG pathways of DEcircRNAs are shown in Figure 3. For the L30-vs-A3Y group, the most significant pathways included the AMPK and PI3K-Akt signaling pathways, while the L30-vs-F90 group was involved in metabolism and myocardial-related pathways. The A3Y-vs-F90 group was the most different from the other comparative groups: a set of pathways was enriched including the TGF-beta, PI3K-Akt, AMPK, and FoxO signaling pathways. These results indicated that circRNAs may play a vital role in muscle development and growth. In particular, the PI3K-Akt signaling pathway was significantly enriched in the three groups, which deserved further investigation.

To elucidate the function of circRNAs, the DEcircRNAs targets were predicted and the circRNA–miRNA network was constructed. We found that 545 of 567 circRNAs harbored one or more miRNA binding sites. The top 10 DEcircRNAs were selected to predict potential target miRNAs and construct an interactive network map (Figure 3D). In the network, several miRNAs related to skeletal muscle development were identified, such as miR-133, miR-125, and miR-181. miR-133 is a well-studied muscle-specific miRNA that is vital for skeletal muscle development as well as the proliferation and differentiation of myoblasts [21,22]. Interestingly, we found that several circRNAs contain at least two conserved target sites for miRNAs related to muscle development. For example, circ-023984 harbors a binding site with miR-133 and miR-125, which are known for regulating muscle growth and development. These circRNAs could serve as candidate circRNAs for further study in sheep skeletal muscle.

### 2.4. Trend Analysis of circRNAs during Muscle Development

**Figure 4:**
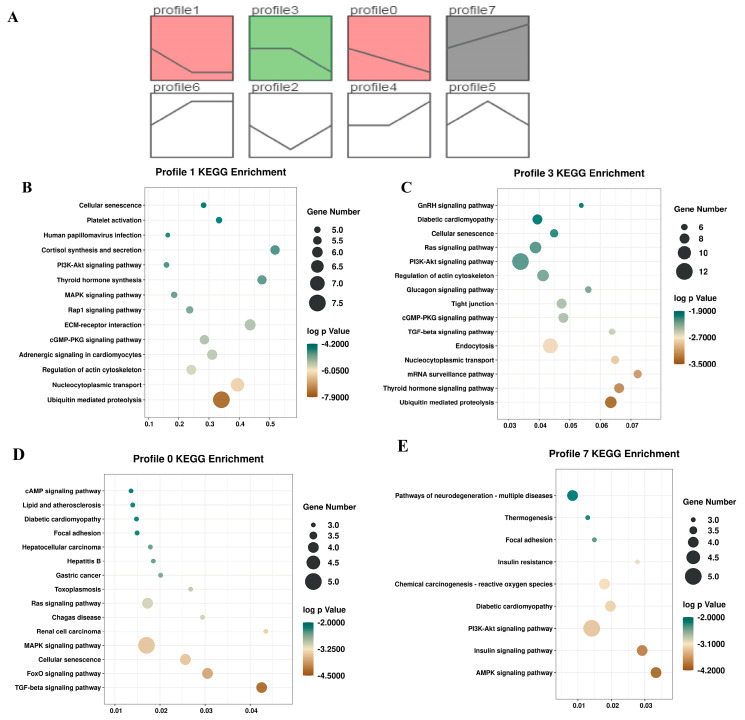
Gene Expression Analysis. (**A**) STEM trend analysis. (**B**) KEGG analysis of the circRNAs in profile 1. (**C**) KEGG analysis of the circRNAs in profile 3. (**D**) KEGG analysis of the circRNAs in profile 0. (**E**) KEGG analysis of the circRNAs in profile 7.

To clarify the circRNA expression trends during muscle development, we performed the STEM trend analysis based on circRNA expression levels. All circRNAs were divided into eight expression patterns, of which four profiles (profile 1, profile 3, profile 0, and profile 7) were significantly enriched (Figure 4A). The heatmap further showed that the four profiles were consistent with the enriched trend (Figure S2). Furthermore, KEGG analysis of circRNAs in the four significantly enriched profiles indicated that the downregulated circRNAs were enriched in the MAPK, TGF-beta, and Ras signaling pathways. For instance, for profile 1, the MAPK, PI3K-Akt, and Rap1 signaling pathways were enriched. In addition, the TGF-beta, AMPK, and Ras signaling pathways were enriched in downregulated profile 0, and profile 7 had an upregulated pattern enriched in the PI3K-Akt and AMPK signaling pathways (Figure 4B–E).

### 2.5. CircCHRNG Promote Myoblast Proliferation

**Figure 5:**
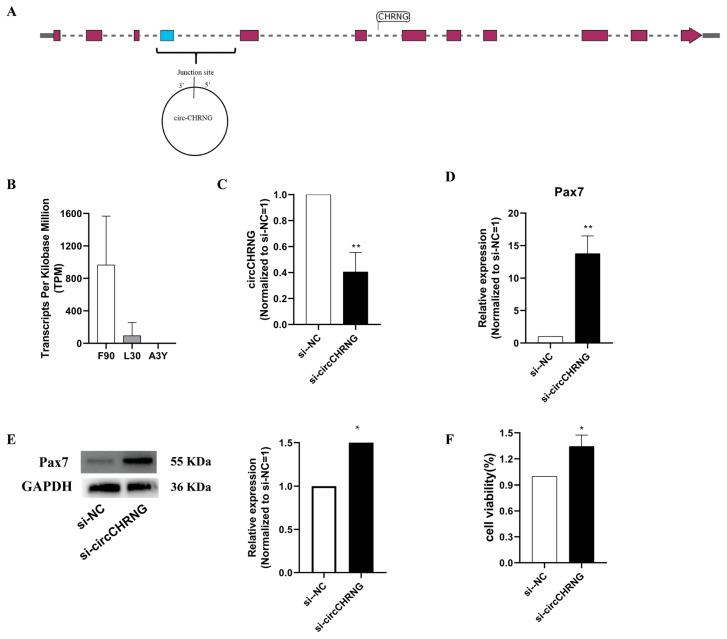
Knockdown of circCHRNG promoted the proliferation of ovine skeletal muscle satellite cells (SMSCs). (**A**) circCHRNG. (**B**) The expression level of circCHRNG in skeletal muscle from F90 to A3Y. (**C**) siRNA of circCHRNG decreased the expression level of circCHRNG. (**D**) Inhibition of circCHRNG increased the mRNA expression level of PAX7. (**E**) Inhibition of circCHRNG increased the protein expression level of PAX7. (**F**) MTT assays for vine skeletal satellite cells transfected with si-circCHRNG or si-NC. *p* < 0.05 was considered to be statistically significant. * *p* < 0.05, ** *p* < 0.01.

As circ-023984 was derived from exon 3 to 4 of CHRNG at chromosome 2, it was termed circCHRNG (Figure 5A). Its expression level decreased sharply during sheep skeletal muscle development (Figure 5B). Furthermore, bioinformatic analysis indicated that circCHRNG harbored binding sites for miR-133 and miR-125, which are known as regulators of myogenesis (Figure 3D). Thus, we selected circCHRNG as a candidate to explore its biological roles in regulating ovine muscle cell myogenesis.

In the present study, ovine skeletal muscle satellite cells (SMSCs, muscle-derived cells) were used as cell models. To further study the potential role of circCHRNG during myoblast proliferation, we designed siRNA and transfected it into ovine SMSCs to inhibit the expression of circCHRNG (Figure 5C). The results showed that knockdown of circCHRNG significantly accelerated the mRNA and protein expression levels of PAX7 during sheep skeletal muscle satellite cell proliferation (Figure 5D,E). Furthermore, the MTT assay result also demonstrated that circCHRNG knockdown promoted myoblast proliferation (Figure 5F). All the results indicated that circCHRNG knockdown significantly promoted myoblast proliferation in vitro.

### 2.6. CircCHRNG interacts with miR-133 and Regulates the Expression of SRF and MEF2A in Myoblasts

**Figure 6:**
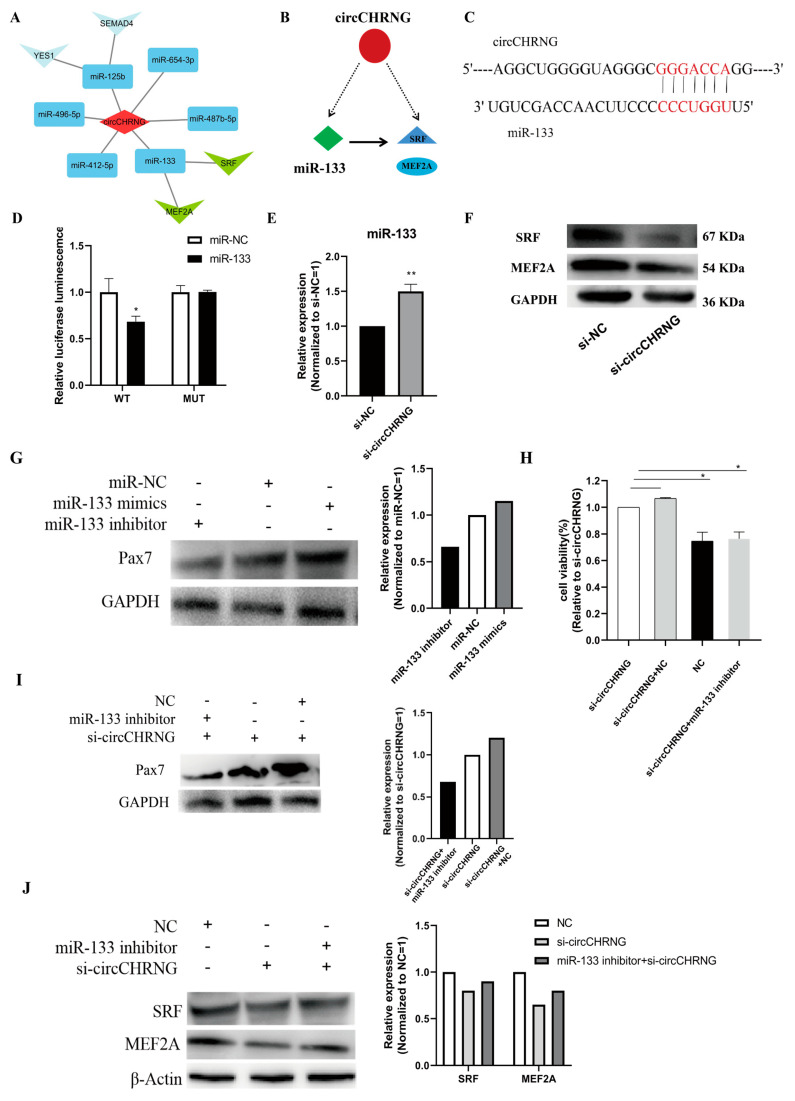
CircRNA–miRNA–mRNA Regulatory Circuitry. (**A**) Interaction network of circCHRNG and miRNAs. (**B**) Illustration of predicted circuitry including miR-133, circCHRNG, and SRF. The red node represents circRNAs; green node represents miRNA; blue node represents key genes related to myogenesis; the solid line represents the real regulatory effect; the dashed line represents the predicted regulatory effect. (**C**) The potential binding sites of miR-133 in circCHRNG. (**D**) circCHRNG wild-type (WT) or mutant (MUT) luciferase reporters and miR-133 mimics, or mimic negative control (NC), were co-transfected in 293T cells. (**E**) Expression of miR-133 in ovine SMSCs transfected with si-NC and si-circCHRNG. (**F**) Protein expression of SRF and MEF2A in ovine SMSCs transfected with si-NC and si-circCHRNG. (**G**) Expression of PAX7 protein in ovine SMSCs infected with miR-NC miR-133 mimics and miR-133 inhibitor. (**H**) MTT assays for ovine SMSCs co-transfected with si-circCHRNG and miR-133 inhibitor or miR-NC. (**I**) Expression of PAX7 protein in ovine SMSCs co-transfected with si-circCHRNG and miR-133 inhibitor or miR-NC. (**J**) Expression of SRF and MEF2A protein in sheep SMSCs co-transfected with miR-133 inhibitor and si-circCHRNG or si-NC. We considered *p* < 0.05 to be statistically significant. * *p* < 0.05, ** *p* < 0.01.

To determine the mechanism of circCHRNG affecting myoblast proliferation, we explored the interactions between miRNAs and circRNAs through a gene interaction network. A regulatory circuitry containing circCHRNG and miR-133 was observed based on bioinformatic analysis, and we found that myocyte enhancer factor-2A (MEF2A) and serum response factor (SRF) were the targets of miR-133 (Figure 6A). Thus, we hypothesized that circCHRNG functions as a miR-133 sponge to indirectly regulate the abundance of SRF and MEF2A and myoblast proliferation (Figure 6B).

To validate the regulatory circuitry, we constructed a dual-luciferase reporter (pmirGLO) by fusing the wild-type (WT) or mutant (MUT) linear sequence of circCHRNG (Figure 6C) with the 3′ end of the firefly luciferase for the luciferase reporter assay. Compared with miR-NC, the firefly luciferase activity of the WT plasmid was significantly reduced by miR-133 (*p* < 0.05), whereas miR-133 did not affect the luciferase activity of the MUT reporter (Figure 6D). In addition, we found circCHRNG inhibition significantly upregulated miR-133 expression (Figure 6E). Altogether, these results suggested a highly efficient interaction between circCHRNG and miR-133.

It has been proved that SRF and MEF2A are direct targets of miR-133, and their expression level can be inhibited by miR-133 [21,23]. In this study, we found knockdown of circCHRNG in ovine SMSCs resulted in a decrease in the protein expression of SRF and MEF2A (Figure 6F). The results indicated that circCHRNG inhibited miR-133 expression and facilitated the expression of MEF2A and SRF.

### 2.7. CircCHRNG Functions as a Sponge of miR-133 to Promote Myoblast Proliferation

miR-133 has been reported to promote myoblast proliferation [24,25]. In our study, the Western blot result demonstrated that overexpression of miR-133 in ovine SMSCs upregulated the expression of PAX7, while miR-133 inhibition decreased the expression of PAX7 (Figure 6G). This confirmed that miR-133 can promote the proliferation of ovine SMSCs, which is consistent with the previous report.

Furthermore, our results indicated that the inhibition of circCHRNG promoted SMSC proliferation (Figure 5D–F), while miR-133 promotes SMSC proliferation and circCHRNG negatively regulates miR-133 expression (Figure 6E); therefore, circCHRNG was likely to affect SMSC proliferation though acting as a sponge of miR-133. To prove this hypothesis, we performed Western blot and MTT assays to examine the expression level of the myogenesis marker gene and cell viability after circCHRNG and miR-133 were co-transfected in the SMSCs. Compared with the control group, the number of SMSC cells remarkedly increased in the circCHRNG inhibition group (*p* < 0.01). No significant difference was observed in the co-transfected (si-circCHRNG and miR-133 inhibitor) group, which can be explained by the miR-133 inhibitor decreasing the cell-proliferation-promoting effect induced by si-circCHRNG (Figure 6H). Similarly, Western blot showed that, compared with the control group, the expression level of PAX7 increased in the si-circCHRNG group (*p* < 0.01) but not in the co-transfected (si-circCHRNG and miR-133 inhibitor) group (Figure 6I). Furthermore, SRF and MEF2A protein expression levels also decreased in the si-circCHRNG group but not in the co-transfected (si-circCHRNG and miR-133 inhibitor) group (Figure 6J). Together, these results suggested that circCHRNG acts as a miR-133 sponge to regulate SRF and MEF2A expression, thus inhibiting myoblast proliferation.

## 3. Discussion

Previous studies mainly focused on the protein-coding genes to explore the molecular mechanisms underlying sheep muscle development and growth. As the function of circRNAs has attracted increasing attention, the characteristics of circRNAs across different tissues in livestock and poultry have been revealed [15,26,27].It has been found that circRNAs are abundantly expressed in the skeletal muscle of domestic animals, some of which are potentially associated with livestock and poultry muscle growth [28]. However, the potential functions and mechanisms of circRNAs in sheep muscle development remained elusive. Our present study provides an overview of the types and relative abundances of circRNAs of sheep skeletal muscle at three developmental stages (fetal, lamb, and adult sheep) using the Ribo-ZeroTM RNA sequencing analysis.

In this study, we investigated the circRNA expression profiles of sheep skeletal muscle during the fetal, lamb, and adult sheep skeletal muscle developmental stages using the Ribo-ZeroTM RNA sequencing method. Compared with other studies, we identified more circRNAs distributed widely across all chromosomes, which reflected the complexity and functional diversity of the circRNAs in muscle [29]. There are obvious differences in the muscle development from embryo to adult sheep. Compared with lamb and adult sheep, more circRNAs were expressed in fetal sheep muscle, suggesting that more circRNAs are involved in the regulation of the proliferation and differentiation of myogenic cells at the embryonic stage. On the other hand, GO analysis showed that the partial function of some circRNAs at the three stages were similar. For example, A band and Z disc were significantly enriched in three groups. A previous study also found A band, I bind, and Z disc were significantly enriched during bovine muscle development [30].

In addition, to further understand the expression trends of DEcircRNAs during the different stages, we also conducted a STEM analysis. The functional enrichment analysis of time-series cicrRNAs in four significant profiles were enriched in pathways associated with muscle development. For example, the upregulated circRNAs in profile 1 were enriched in the AMPK and PI3K-Akt signaling pathways, while the downregulated circRNAs were enriched in the MAPK and TGF-beta signaling pathways during the three development stages. Moreover, KEGG analysis showed that the DEcircRNAs were significantly enriched in some signaling pathways, including the Jak-STAT, TGF-beta, and PI3K-Akt signaling pathways, which regulate cell proliferation, invasion, and differentiation, as well as other essential cellular processes [31,32]. Altogether, our analysis suggested that the PI3K-Akt signaling pathway and TGF-beta have vital roles in muscle development across the fetal, lamb, and adult sheep stages.

It has been reported that circRNAs may regulate muscle development by functioning as miRNA sponges [29,33]. In order to explore the role of circRNAs in sheep muscle growth and development, a circRNA–miRNA network was established to predict the potential relationship between circRNAs and miRNAs. Our results indicated that some circRNAs (circ_020954, circ_020457, circ_013215, cric_032546, and circ_006116) contained binding sites of miRNA-29. Previous studies revealed that circHIPK3 targets miR-29 and acts as a miR-29 sponge to regulate cardiac fibroblast growth and lung fibroblast-to-myofibroblast transition [34,35]. Moreover, circRNAs also target some myogenesis miRNA, such as miR-181, miR-125, and miR-133 [36,37]. For instance, miR-125 displays increased expression during both cardiac muscle development and iPS-derived cardiomyogenesis [37], and miR-125 inhibits vascular smooth muscle cell proliferation [38]. miR-181 was strongly upregulated during differentiation; it regulates skeletal muscle differentiation by targeting the homeobox protein Hox-All [39]. miR-133 plays an important role in the regulation of skeletal muscle proliferation and differentiation [21,40], and miR-133 post-transcriptionally represses IGF-1R expression during myogenic differentiation of C2C12 by directly binding to its 3′UTR and thus negatively modulating the PI3K-Akt signaling pathway [41]. Therefore, these circRNAs may act as key regulators of skeletal muscle growth as potential ceRNAs.

Recent evidence has revealed that the expression of circRNAs plays an important role in muscle development [14,42], and the functional circRNAs often show developmental stage-specific expression patterns [43]. In the present study, we identified a novel cricRNA, named circCHRNG, and found that the expression of circCHRNG was remarkably reduced from fetal to adult sheep muscle. It is worth mentioning that the circCHRNG [44] host gene is associated with the signal transduction of neural cells with skeletal muscle cells and participates in the neuroactive ligand–receptor interaction signal pathway. From these characteristics, we speculated that circCHRNG might be involved in regulating muscle development through signal transduction pathways. Therefore, we sought to determine the biological function of circCHRNG in muscle development. In vitro cellular experiments indicated that knockdown circCHRNG could promote ovine SMSC proliferation. In recent years, much evidence has shown that circRNAs serve as a miRNA sponges to regulate muscle growth [42,45]. We hypothesized that circCHRNG might sequester miRNAs, thereby effecting muscle development. In order to verify this, the possible target miRNAs of circCHRNG were predicted by bioinformatics analysis. To verify whether miR-133 is regulated by circCHRNG, dual-luciferase reporter assay and qPCR were performed, and miR-133 was downregulated by circCHRNG in ovine SMSCs. Earlier studies have reported that miR-133 may act as a skeletal-muscle-specific miRNA involved in C2C12 proliferation and differentiation [36,46]. Here, miR-133 was also found to positively regulate sheep SMSC proliferation. The inhibition of miR-133 rescued the function of circCHRNG knockdown. Taken together, these results demonstrated that circCHRNG acts as a sponge of miR-133 to regulate the proliferation of ovine SMSCs.

Previous studies have proved that SRF or MEF2A regulate myoblast proliferation. In mice, miR-133 enhances myoblast proliferation by repressing serum response factor (SRF) [21]. In bovine animals, the overexpression of myocyte enhancer factor-2A (MEF2A) inhibits myoblast proliferation by triggering cell cycle progression [47]. In C2C12, MEF2A knockout mice display impaired regenerative myogenesis [48]. Moreover, the muscle-specific miR-133 affect the expression of SRF and MEF2A [21,23]. According to our results, the protein expression levels of SRF and MEF2A were decreased and promoted the proliferation of ovine SMSCs in silent circCHRNG. We speculate that circCHRNG may affect SRF and MEF2A expression and thereby regulate SMSC proliferation through sponging miR-133. Although we confirmed that circCHRNG serves as a sponge for miR-133 to modulate the proliferation of SMSCs, the pathways involved in regulating muscle cell proliferation induced by circCHRNG need to be further elucidated. In addition, the function of circCHRNG needs be investigated in vivo, which could provide more powerful evidence for the molecular mechanism of muscle development in ovine animals.

## 4. Materials and Methods

### 4.1. Experimental Samples and Ethics Statement

Duolang sheep, a Chinese local breed, is characterized by a fast growth rate, high meat yield, high reproductive rate, and early maturing. Healthy Duolang sheep were raised under the same conditions of free access to water and food in Changping experimental base in Beijing, China. The longissimus dorsi samples of 90-day-old fetus (F90), 30-day-old lamb (L30), and three-year-old adult sheep (A3Y) were collected. All tissue samples were immediately frozen in liquid nitrogen after harvesting.

All animal experimental procedures were approved by the Ministry of Agriculture of the People’s Republic of China and the Institute of Animal Science, Chinese Academy of Agricultural Sciences and were performed according to the guidelines for the care and use of experimental animals established by this ministry. Ethical approval for animal survival was provided by the animal ethics committee of the Institute of Animal Science, Chinese Academy of Agricultural Sciences (IAS-CAAS) with the following reference number: IASCAAS-AE-03, on 1 September 2014.

### 4.2. Library Construction and Sequencing

Total RNA was isolated from nine longissimus dorsi samples using RNeasy^®^ Plus Universal Mini Kit (Qiagen, Germany) according to the manufacturer’s instructions. The purity and quantity of total RNA were determined using agarose gels, the Agilent Bioanalyzer 2100 (Agilent Technologies, Santa Clara, CA, USA), and the RNA 6000 Nano Kit (Agilent Technologies, Santa Clara, CA, USA), and the qualified RNA was used to construct the cDNA library. Firstly, ribosomal RNA was removed from 3 ug of total RNA according to the protocol of the Ribo-ZeroTM Magnetic Gold Kit (Epidemiology, Madison, WI, USA). Then, RNase R (Epicentre, WI, USA) was used to remove linear RNA. Next, the RNA was randomly broken into fragments and the polymerase chain reaction (PCR) was performed to construct the cDNA library. The libraries were sequenced on the Hiseq2500 platform (Illumina, San Diego, USA) using the 150 bp paired-end (150PE) mode.

### 4.3. Identification of Circular RNAs in Sheep Muscle

Clean reads were obtained by removing reads containing adapter and low-quality reads (reads with a rate of N greater than 10% and reads with the base number of mass value Q ≤ 10 accounted for more than 50% of the whole reads) from raw reads. The clean reads were mapped to the sheep reference genome using the software BWA (v0.7.13). The sheep reference genome (Oar-v4.0) and gene annotation file were downloaded from NCBI (http://www.ncbi.nlm.nih.gov accessed on 27 December 2020). The CIRI (V2.0.5) based on the BWA-MEM algorithm was used to identify circRNAs. Only those containing more than two independent junction-spanning reads and corresponding to the GU/AG rules were determined as candidate circRNAs.

### 4.4. Differential Expression Analysis

The expression level of circRNAs was normalized by transcript per million (TPM). The differential expression analysis between two groups was assessed using DEGseq2 packages (1.10.1). CircRNAs with a false discovery rate (FDR) of < 0.01 and an absolute value of log2 (fold change) of > 2 were assigned as differentially expressed circRNAs.

### 4.5. Target Site Prediction and Functional Enrichment Analysis

The target miRNAs of differentially expressed circRNAs were analyzed using miRanda (v.3.3a). A circRNA–miRNA interaction network was constructed with Cytoscape (v.3.8.0). The GO (Gene Ontology) and KEGG (Kyoto Encyclopedia of Genes and Genomes) enrichment analyses of the host genes of circRNAs were performed with the Gene Ontology Resource (http://geneontology.org/, accessed on 30 August 2021) and KOBAS software, respectively. Scores with *p* < 0.05 were considered significant for enrichment analysis.

### 4.6. Data Validation

To assess the expression patterns deduced from the sequencing data, six circRNAs were randomly selected for RT-qPCR analysis. cDNA was synthesized using the PrimeScript TM RT reagent kit with the gDNA Eraser kit (Takara, Dalian, China). The qPCR was performed on an ABI 7500 (Applied Biosystems, Waltham, MA, USA) using the SYBR Premix Ex Taq Π kit (Takara, Dalian, China) according to the manufacturer’s protocol. Thermal cycling consisted of an initial step at 95 °C for 10 min followed by 40 cycles at 95 °C for 30 s and 62 °C for 30 s. The levels of the circRNAs were determined relative to the expression levels of β-actin. RT-qPCR was performed using the following reaction system: 10 µL of SYBR Premix DimerEraser, 2 µL of cDNA, 0.4 µL of Rox Reference Dye, 0.8 µL of upstream and downstream primers, and 6 µL of ddH_2_O. The levels of circRNAs digested by RNase R were normalized to levels of circRNAs not digested. Linear mRNA of GAPDH (sensitive to RNase R) was selected as a positive control. The qPCR measurements were performed in triplicate for each cDNA sample and the GAPDH gene was used as a reference gene. The relative expression levels of the circRNAs were determined with the 2^−ΔΔCT^ method based on the cycle threshold (Ct) values. The primers are detailed in Table S1.

### 4.7. Cell Culture and Transfection

Sheep skeletal muscle satellite cells (SMSCs) were isolated from the leg muscles of F90. Leg muscle (1 g) was minced into sections of approximately 1 mm^2^ with scissors and digested with 0.25% trypsin (Gibco, Grand Island, NY, USA) at 37 °C in a shaking water bath (90 oscillations/min). Digestions were terminated by adding fetal bovine serum (Gibco) after 30 min. The mixture was filtered through a nylon mesh with 70 mm pores (BD Falcon). The filtered cells were centrifuged at 350× *g* for 5 min, and the isolated cells were cultured in DMEM/F12 (Ham) (Gibco) with 20% fetal bovine serum and 0.2% penicillin/streptomycin.

The wild-type and mutated sequences of circCHRNG were synthesized and cloned into the pmirGLO dual-luciferase reporter vector (Promega) with the NheI and XhoI restriction sites. Cells were transfected with 50 nM of miRNA mimics and 100 nM of siRNA or plasmid (1 µg/mL) using Lipofectamine 3000 reagent (Invitrogen) according to the manufacturer’s instructions (Table S2).

### 4.8. Dual-Luciferase Reporter Assay

The sequences of circRNA 2:233240382|233241086 were located on chromosome 2 and derived from cholinergic receptor nicotinic gamma subunit (CHRNG), and thus we termed it circCHRNG. circCHRNG and its corresponding mutant without oar-miR-133 binding sites were synthesized and subcloned into pmirGLO dual-luciferase reporter plasmid. The miR-133 mimics and negative control (NC) were co-transfected into cells with 3′-UTR dual-luciferase vector using Lipofectamine 3000 (Invitrogen). Cells were collected 24h after transfection, and the relative luciferase activity was examined using the Dual-Luciferase Assay Kit (Promega, Madison, WI, USA) in accordance with the manufacturer’s protocols. Three replicates were performed for each transfection.

### 4.9. Cell Proliferation Assays

The cell viability upon transfection of siRNA was assessed by MTT assay (Solarbio, Beijing). After knockdown, the circCHRNG and the proliferation of myoblasts was tested using MTT assay (Solarbio, Beijing). The cells were exposed to 10 µL MTT for 4 h at 37 °C following the manufacturer’s instructions. The viability of the cells was quantified as the percentage (%) of living cells relative to untreated cells.

### 4.10. Western Blot Analysis

Total proteins from cells were homogenized using RIPA buffer. Protein concentrations were determined using the BCA Protein Assay Kit (Thermo Pierce, United States). Proteins were separated with 10% SDS-PAGE, transferred onto a PVDF membrane (Bio-Rad, Hercules, CA, USA) and blocked in 5% nonfat milk. The membrane was incubated with primary antibodies: anti-β-Actin (Proteintech, 66009-1-Ig, 1:4000), anti-Pax7 (Bioss, bs-2413R, 1:1000), anti-SRF (CST, 5147S, 1:1000), anti-MEF2A (Bioss, bs-5485R, 1:1000), and anti-GAPDH (Proteintech, HRP-60004, 1:4000) overnight at 4℃. The membranes were immunoblotted with goat anti-rabbit (Solarbio, SE13 1:5000) or goat anti-mouse (Solarbio, SE131 1:5000) at room temperature for 1h, and an enhanced chemiluminescence kit (Fdbio) was used to visualize specific protein bands as previously described.

## 5. Conclusions

In summary, our study provides a systematic description of circRNAs related to ovine muscle development during the fetal, lamb, and adult stages. GO and KEGG enrichment analyses showed that DEcircRNAs were enriched in multiple pathways associated with muscle development, such as PI3K-Akt, AMPK, and Jak1. Furthermore, the visual circRNAs–miRNAs transcription regulatory networks generated in this study provide a valuable resource of candidate circRNAs involved in myogenesis. In addition, we propose that circCHRNG acts as a miRNA (miR-133) sponge to regulate the abundance of SRF and MEF2A, thereby regulating the proliferation of skeletal muscle satellite cells. Our study provides useful information on the functional circRNAs in sheep muscle which can be applied to the improvement of meat production in future studies.

## Data Availability

The datasets used and analyzed during the current study are available from the corresponding authors on reasonable request. The raw reads produced in this study were deposited in the NCBI Sequence Read Archive (SRA), and the records can be accessed by accession number PRJNA883616.

## Supplementary Materials

The following supporting information can be downloaded at: https://www.mdpi.com/article/10.3390/ijms232416065/s1.
