# Supplementary material for: Genome-Wide Analysis of Circular RNAs Reveals circCHRNG Regulates Sheep Myoblast Proliferation via miR-133/SRF and MEF2A Axis
Source: Int J Mol Sci. 2022 Dec 16;23(24):16065. doi: 10.3390/ijms232416065 (PMC9781509; doi:10.3390/ijms232416065)
Supplement: Supplementary file 1 [file ijms-23-16065-s001.zip › ijms-2066539-supplementary.pdf]

Supplementary Material

Supplementary Tables

Table S1: Primers sequence for real-time quantitative PCR.

Gene	Forward Primer Sequence（5'→3'）	Reverse Primer Sequence（5'→3'）
β-Actin	CCAACCGTGAGAAGATGACC	CCAGAGGCGTACAGGGACAG
U6	GTGCTCGCTTCGGCAGCACATAT	AAAATATGGAACGCTTCACGAA
GAPDH	CAGAACATCATCCCAGCGT	CAGGTCAGGTCAACAACAG
circOBSCN	TTCCGTGGCCTCCTCCTT	CCCTGCCTGCCAAGTTCC
circGFM1	ATCTTCCACTTAGCCTCAT	CTACAATGTTCCGTTTCTCACTTTC
circCDYL	AGTATTCCGTCTTCCCTTTCTTGTT	CGTGAATGGGAAAGGTTGAA
circLRIG1	CGGGGAGGTGATGGAGTA	CCACGGCTGTGATGGTCT
circTTN	CAGTGATGCCTCCAAAGC	GGGGTCACAGCGTCCAGGAGGGTCT
circCHRNG	TGGATTTCTGCCTCCCTAT	TTGCTGCATCTGAGCTTTCT
circMDN1	GCCTGTTTCTGTCCTGCTAC	CGCTCTTCCTCTTCCTCCTCGCTCA
circLOC1	TCAAGACGGGTCGGGTGGGT	AGCCGGCGGCCGAGCGCACGGGGTC
miR-133	ACACTCCAGCTGGGCAAAGTGCTTAC AGTGC	CTCAACTGGTGTCGTGGA
SRF	CCCTCCTTTCCCATCACC	GCCGCTGCCTGTACTCTT

Table S2: Sequences of siRNA.

Sequence name	sense（5'-3'）	antisense（5'-3'）
si-circCHRNG	GGAUAGAAAUGGCAGUGGUTT	ACCACUGCCAUUUCUAUCCTT
miR-133 inhibitor	ACAGCUGGUUGAAGGGGACCAA	
miR-133 mimics	UUGGUCCCCUUCAACCAGCUGU	AGCUGGUUGAAGGGGACCAAUU
mimics NC	UUCUCCGAACGUGUCACGUTT	ACGUGACACGUUCGGAGAATT

Table S3: Summary of the RNA-seq data for each replicate.

Sample	Read Sum	Base Sum	GC (%)	Q20 (%)	Q30 (%)	reads_mapped
F90-1	46,709,908	11,677,477,000	49.34%	94.56%	89.94%	93312756 (99.89%)
F90-2	40,790,217	10,197,554,250	49.64%	93.21%	87.70%	81312458 (99.67%)
F90-3	56,914,493	14,228,623,250	49.38%	93.33%	87.88%	113731985 (99.91%)
L30-1	47,787,038	11,946,759,500	48.27%	92.82%	87.04%	95472106 (99.89%)
L30-2	50,962,735	12,740,683,750	47.85%	94.83%	90.35%	101845813 (99.92%)
L30-3	52,134,934	13,033,733,500	48.47%	93.19%	87.60%	104125500 (99.86%)
A3Y-1	49,649,352	12,412,338,000	50.01%	92.82%	87.02%	99165738 (99.87%)
A3Y-2	56,134,489	14,033,622,250	49.19%	93.87%	89.00%	112146866 (99.89%)
A3Y-3	50,391,945	12,597,986,250	49.59%	93.80%	88.87%	100701969 (99.92%)

Supplementary Figures S1

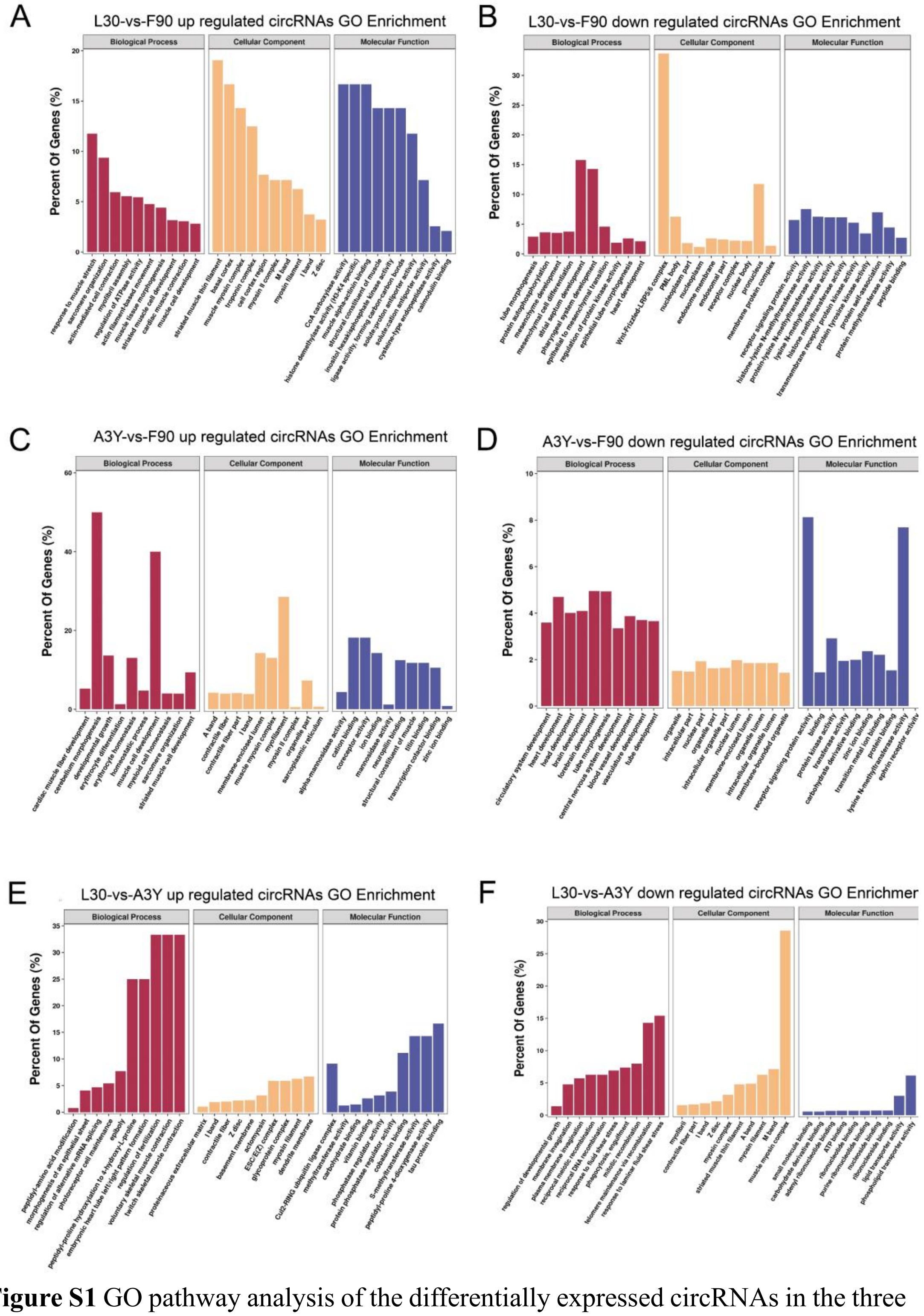

Figure S1 GO pathway analysis of the differentially expressed circRNAs in the three group. **(A)** Histogram of up regulated differential circRNAs GO enrichment in the L30-vs-F90. **(B)** Histogram of down regulated differential circRNAs GO enrichment in the L30-vs-F90. **(C)** Histogram of up regulated differential circRNAs GO enrichment in the A3Y-vs-F90. **(D)** Histogram of down regulated differential circRNAs GO enrichment in the A3Y-vs-F90. **(E)** Histogram of up regulated differential circRNAs GO enrichment in the L30-vs-A3Y. **(F)** Histogram of down

regulated differential circRNAs GO enrichment in the L30-vs-A3Y.

Supplementary Figures S2

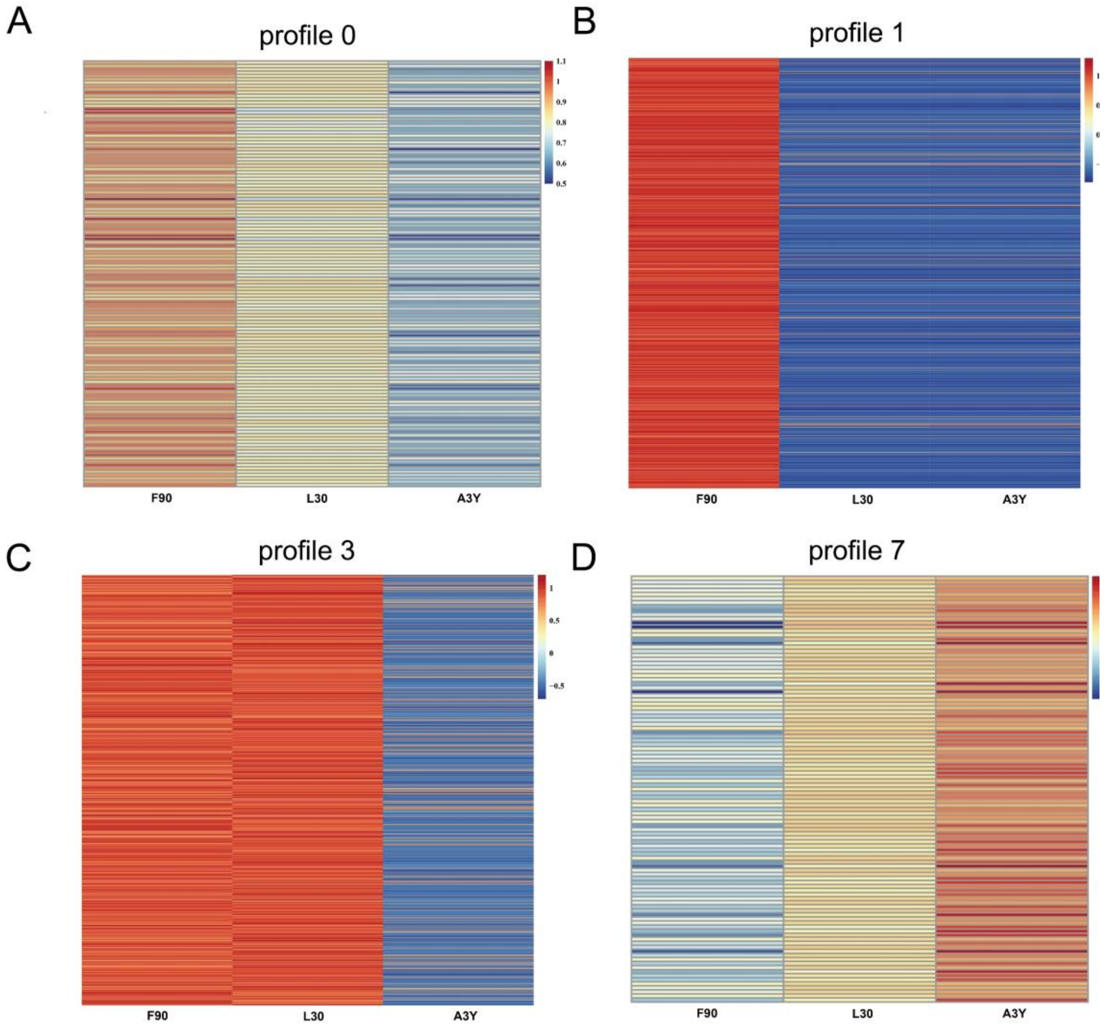

Figure S2 Hierarchical clustering plot of cicrRNAs expression in significantly different profile. **(A)** The hierarchical clustering of circRNAs in Profile 0. **(B)** The hierarchical clustering of circRNAs in Profile 1. **(C)** The hierarchical clustering of circRNAs in Profile 3. **(D)** The hierarchical clustering of circRNAs in Profile 7.
